# Specific relations of visual skills and executive functions in elite soccer players

**DOI:** 10.3389/fpsyg.2022.960092

**Published:** 2022-08-25

**Authors:** Antonia Knöllner, Daniel Memmert, Marec von Lehe, Johannes Jungilligens, Hans-Erik Scharfen

**Affiliations:** ^1^Department of Neurosurgery, Brandenburg Medical School, University Hospital Ruppin-Brandenburg, Neuruppin, Germany; ^2^Institute of Exercise Training and Sport Informatics, German Sport University Cologne, Cologne, Germany; ^3^Department of Neurology, University Hospital Knappschaftskrankenhaus Bochum, Ruhr University Bochum, Bochum, Germany; ^4^Neurocognition and Performance Lab, SV Werder Bremen, Bremen, Germany

**Keywords:** visual functions, cognitive functions, executive functions, high-performance athletes, near-far quickness, working memory, football

## Abstract

Visual and cognitive skills are key to successful functioning in highly demanding settings such as elite sports. However, their mutual influence and interdependencies are not sufficiently understood yet. This cross-sectional study examined the relationship between visual skills and executive functions in elite soccer players. Fifty-nine male elite soccer players (age: 18–34 years) performed tests assessing visual clarity (left-, right-, and both eyes), contrast sensitivity, near-far quickness, and hand-eye coordination. Executive function measures included working memory capacity, cognitive flexibility, inhibition and selective attention. Overall, visual abilities were largely correlated with executive functions. Near-far quickness performance showed a large correlation with an executive function total score as well as with cognitive flexibility, working memory, and especially selective attention. Visual clarity and contrast sensitivity were moderately correlated with the cognition total score. Most consistent correlations with the visual functions were present for working memory. These findings present an overall vision-cognition relationship but also very specific linkages among subcategories of these functions, especially meaningful relations between near-far quickness, selective attention and cognitive flexibility. Further studies are needed to investigate the neuropsychological mechanisms accounting for the correlations and possible improvements of the executive functions by training specific visual skills.

## Introduction

Professional elite team sports demand athletes with extraordinary cognitive (Scharfen and Memmert, 2019a) and visual skills (Burris et al., 2018). Therefore, the interest to understand, examine, and improve these skills has increased rapidly within the last years (Walton et al., 2018; Formenti et al., 2019). However, behavioral data on the linkage between subcategories of vision and cognition are rare and a precise understanding of the relation is still unclear (Vu et al., 2021). Regarding the cognitive domain, cognitive functions are described as a concept that involves mental activities including knowledge acquisition, information processing, reasoning, and the domains of perception, attention, learning, memory, decision making, and language abilities (Kiely, 2014). An essential subcategory of cognitive capabilities are executive functions that cover cognitive abilities that regulate thought and behavior in non-routine situations. Thus, enabling mental playing with ideas, thinking before acting, coping with novel and unexpected challenges, resisting temptations, and staying focused (Friedman et al., 2006; Diamond, 2013). These executive functions are divided into core and higher-level executive functions. The former comprise (i) inhibitory control: the ability to control one’s attention, behavior, thought, and emotion to overcome a strong internal or external lure for executing a more appropriate action; (ii) working memory: mentally working on information, that is held in mind; (iii) cognitive flexibility: the ability to shift attention between task sets or strategies during problem-solving, thinking “outside the box” with creativity (Miyake et al., 2000; Diamond, 2013).

A recent meta-analysis indicates, that the executive function performance of elite athletes are superior compared to amateur or non-athletes. More precisely, executive functions show relevant associations with several soccer performance related aspects including motor abilities (Scharfen and Memmert, 2019b; Reigal et al., 2020a), game performance (Sabarit et al., 2020; Scharfen and Memmert, 2021b), psychosocial functioning (Reigal et al., 2020b), game intelligence (Scharfen and Memmert, 2021b).

Concerning the visual domain, visual information is of major importance for executive functions since information gathered by the eyes is processed in the brain to create a precise image of the environment. In particular, vision represents one of the evolutionary most important sensory information sources (Marteniuk, 1976). The crucial role of vision in the neuronal hierarchy is underlined by the fact that a large amount of cortical and subcortical structures are involved in vision and visual processing (Kandel et al., 2000; Goldstein, 2010; Bear et al., 2015; Sternberg and Sternberg, 2016). Furthermore, vision influences the other somatosensory inputs (Taylor-Clarke et al., 2002; Cardini et al., 2011). Optimal visual perception requires several visual abilities, including (i) visual clarity: the ability to process non-moving visual information while standing still; (ii) contrast sensitivity: the ability to perceive temporal or spatial information about objects and their backgrounds under changing light conditions; (iii) near-far quickness: the ability to quickly change gaze focus between far and near distances (Erickson et al., 2011).

The relationship between vision and cognition is of major interest in elite sports as perceptual and cognitive skills are of fundamental relevance for elite performance (McGuckian et al., 2020). Specifically, distinct vision-cognition linkages were proposed with considerable mutual influences in shared underlying brain structures such as the superior colliculi (Basso et al., 2021). The premotor theory of attention states that neurons involved in preparing eye movements are also involved in controlling shifts in attention through a network of subcortical structures like the superior colliculi (Basso et al., 2021). Therefore, these structures seem to impact not only visual skills but also executive functions like selective attention and cognitive flexibility since they strongly depend on shifts in attention (Diamond, 2013). According to this theory, visual skills that include controlling gaze focus like near-far quickness might have a strong relation to selective attention and cognitive flexibility.

Recently, the essential impact of subcortical structures on executive functions has been proposed. Specifically, the lateral geniculate nucleus of the thalamus has traditionally been described as a gateway for sensory information entering the visual cortex. But furthermore, it has been shown that the lateral geniculate nucleus receives input also from cortical structures. In particular, selective attention and visual awareness can modulate the neuronal activity within the lateral geniculate nucleus in multiple ways (O’Connor et al., 2002; Saalmann and Kastner, 2011). Moreover, primitive visual pathways, e.g., visual spatial perception present a causal role in cognitive transfer and higher cognitive processes (Saban et al., 2021), e.g., mathematical problem solving (Sella et al., 2016). Regarding the multicomponent model of working memory a so-called visuo-spatial sketchpad serves as short-term storage for visual information (Baddeley, 2010; Diamond, 2013). Therefore, a direct relationship between working memory- and the visual system is proposed from that theoretical standpoint.

However, behavioral performance data on the vision-cognition interaction in elite athletes do not exist yet and it is unclear which specific visual and executive functions are related to each other. Therefore, the aim of the present study is to analyze the relationship of specific subgroups of visual and executive functions aiming to set a starting point for further research in this area to show possible options for performance improvements and scouting in elite sports. To the best of our knowledge, this is the first study investigating this interrelation. We are particularly following the call of Vu et al. (2021) to examine specific visual subgroups. These include visual acuity, near-far quickness, contrast sensitivity, and hand-eye coordination since these visual skills are the most important ones for soccer players (Roberts et al., 2017; Burris et al., 2020). Also, the executive functions investigated in this study were chosen due to their fundamental relevance to elite soccer: working memory, inhibition, cognitive flexibility, and selective attention (executive functions; Vestberg et al., 2012, 2017; Verburgh et al., 2014; Huijgen et al., 2015; Scharfen and Memmert, 2021b; selective attention; Faubert, 2013; Romeas et al., 2019). Based on previous literature it is firstly hypothesized that the examined visual and executive functions are positively correlated. Secondly, it is hypothesized that the strongest correlations are present for the visual function near-far quickness with the executive functions of selective attention and cognitive flexibility. Thirdly, correlations between working memory and all visual functions are hypothesized.

## Materials and methods

### Participants

Data of 59 male elite soccer players of a German team playing in the first division of the German Bundesliga in season 2020/2021 (*n* = 26) or second division of the German Bundesliga in season 2021/2022 (*n* = 7) or a U23 team, playing in the fourth-highest division of the German Bundesliga, in season 2020/2021 (*n* = 26) were analyzed in this study. The participants had a mean age of 22.81, ranging from 18.05 to 34.87 years of age. The data acquisition of this cross-sectional study was part of the annual pre-season performance diagnostic of the seasons 2020 (*n* = 52) and 2021 (*n* = 7). Only data of players who did not engage in the 2020 testing were included in the 2021 data analysis, hence the smaller sample size in 2021. The performance tests of the pre-season diagnostic 2021 did not include the contrast sensitivity test; accordingly, this data is not present for seven participants. None of the participants was diagnosed with any behavioral, learning, or medical condition that might influence cognitive abilities. The study was carried out accordingly to the Declaration of Helsinki of 1975 and was approved by the ethics committee of the German sports university cologne with the protocol number 085/2020. Executive functions are analyzed in terms of working memory, cognitive flexibility, inhibition, and selective attention whereas visual function measures included visual clarity, contrast sensitivity, near-far quickness, and hand-eye coordination.

### Materials

#### Executive function tests

*Working memory capacity (WMC)* was assessed with the working memory span test of Conway et al. (2005). This test assesses the player’s ability to direct attention toward the present task without getting distracted. Therefore, a counting span task was applied (Kane et al., 2004). Instructions of the test were displayed as written text on the computer screen. In the counting span task, the participants were asked to count specific shapes, dark blue circles, among distractor shapes, green circles, and dark blue squares, and remember the count totals for a later recall. In every stimulus image, the shapes were randomly arranged. After 2–7 stimulus images a recall mask was presented, in which the participants were asked to fill in their memorized count totals in the same order as they had been presented to them. The participants counting span score was a partial credit load score (Conway et al., 2005), which represents the sum of all correctly recalled elements—whereby a correctly remembered piece from a set containing two elements receives 2 points, and a correctly remembered element from a set with 7 items receives 7 points—divided by the maximum possible score. Good reliability and validity are stated for the test (ICC = 0.7–0.9; Conway et al., 2005). The test included 15 trials. The dependent measure was the score of correctly memorized objects in percentage representing the WMC (Scharfen and Memmert, 2019b).

*Cognitive flexibility* was measured with the trail-making test (TMT) consisting of two parts (A and B) (Sánchez-Cubillo et al., 2009). The TMT-A is generally used to assess visuoperceptual ability whereas the performance time difference of TMT-B minus TMT-A is used to determine cognitive flexibility. A smaller B-A difference implies better cognitive flexibility (Crowe, 1998). In part A (TMT-A), the test subject had the task to connect encircled numbers from 1 to 25 in ascending order as fast as possible. In addition to the numbers, part B (TMT-B) comprises letters. Participants were required to connect numbers, 1–13, and letters, A to L, in a numerical and alphabetical ascending order, whereby numbers and letters had to be alternating (1-A-2-B-3-C…). The time that each participant needed to complete each of the subtests and the number of mistakes were recorded. In the case of performance errors, the instructor directly announced them and asked the participant to correct them immediately, so they are included in the overall time. To ensure an adequate understanding of the task requirement, a practice trial of each part was completed before the measurements, consisting of the numbers 1–8, respectively, the numbers 1–4, and the letters A to D. A tablet version of the TMT was used, which has been shown to be congruent with the traditional pen-paper version (Delbaere and Lord, 2015; Baykara et al., 2022). General validity and reliability of the TMT have been shown (Smith et al., 2008; Wagner et al., 2011).

*Inhibitory control* was assessed with a computer-based language-independent stop-signal task (SST) from the Cambridge Neuropsychological Test of Automated Battery (CANTAB; Cambridge Cognition, 2019). The response inhibition was measured by conducting two opposing tasks: a Go task and a Stop task. For the Go task the participants were instructed to press a left-hand button as quickly as possible when an arrow pointing to the left appeared, and a right-hand button, when an arrow pointing at the right appeared (Go task in 75% of trials). Additionally, for the Stop task, they were assigned to inhibit the response and not press any of the buttons when they heard an auditory “beep” signal (Stop task in 25% of trials). The onset of the “beep” signal altered in dependence on the participant’s performance within the previous trials, accordingly to a staircase protocol (i.e., either decreased or increased). As the dependent variable the stop-signal reaction time (SSRT) which is an estimate of the time a participant needed to stop his or her response minus the mean delay was used. Shorter SSRTs are indicating better inhibitory control (Matzke et al., 2018). Detailed information about this test protocol was described previously (Matzke et al., 2018). It has been indicated that the SSRT is highly reliable (Williams et al., 1999).

*Selective attention* was assessed in a Multiple-Object Tracking, precisely in the NT 3D-MOT task with the NeuroTracker™ Core Program by CogniSens Athletics Inc. from the University of Montreal. The program was displayed on a wall with the help of a video projector. NT 3D-MOT settings were set up as described in Faubert (2013). During the task, eight yellow balls, of which four changed their color after 1 s to orange, were presented. Participants were asked to memorize the indicated balls. Then, all balls moved randomly through the 3D domain with a specific velocity for 8 s. After 8 s, the balls stopped moving, and the participant‘s task was to indicate the four balls (targets), that have changed their color to orange in the beginning. Afterward, participants received feedback, and the next trial started. The moving velocity of the balls in the following trial was dependent on the participant’s performance in the previous trial. One session consisted of 20 trails, overall lasting about 8 min. The dependent measure was the average speed threshold among all trials (for detailed information see Faubert, 2013).

The overall executive function score (executive function total) was calculated by adding the z-standardized scores of all cognitive tests, (1) working memory capacity, (2) cognitive flexibility, (3) inhibition, (4) selective attention, and dividing the sum by the number of included tests.

#### Visual tests

The Senaptec Sensory Station was used to assess contrast sensitivity, near–far quickness, and hand-eye coordination, with proven reliability (for a detailed description of the procedure and reliability; see Erickson et al., 2011). Visual clarity was measured with a Snellen chart accordingly to the standard procedure (Azzam and Ronquillo, 2022). Visual clarity was separately measured for the left, the right and both eyes. In contrast, the other tests were performed with both eyes. Dependent measures of the individual visual functions are described below:

*Visual clarity*, the ability to process non-moving visual information while standing still, was measured with the help of a Snellen chart which depicts different letters in descending size. The participants stood at a 6.1-m distance from the Snellen chart and were asked to read the letters from top to bottom out loud, each with the right, left, and both eyes. As soon as two errors were made in a row, these corresponded to the final score—dependent variable: threshold of the static visual acuity.

*Contrast sensitivity* is described as the ability to perceive temporal or spatial information about objects and their backgrounds under changing lighting conditions. The test protocol was the following: four black circles, arranged in a diamond shape were presented to the participants on a screen. The participants stood at a distance of 4.9 m from the screen. During the test, one of the circles was randomly marked by the appearance of smaller circles of different brightness within the certain circle. The participant’s task was to indicate the marked cycle by swiping on a smartphone in the corresponding direction. The measured dependent variable was the threshold sensitivity between 10 and 1%.

*Near-far quickness*, the ability to quickly switch the gaze between far, intermediate, and near distances requiring rapid accommodative-vergence responses. The participants were placed at a 4.9 m distance to the screen, holding a smartphone at a distance of 40 cm from themselves in their hands. Landolt rings, circles with an orifice either at the top, bottom, left or right, were presented to the participants, alternating on the screen and the smartphone. The size of the Landolt rings was adjusted to the individuals’ visual clarity. The task was to indicate as many openings of the Landolt rings correctly as possible within 30 s by swiping in the corresponding direction on the smartphone. The number of correctly indicated Landolt rings was measured.

*Hand-eye coordination* is defined as the ability to process and respond to visual stimuli. Circles were presented to the participants on a screen. The circles were the same size and evenly distributed in a grid of 8 columns and 6 rows. During the test, one of the circles was indicated by lighting up yellow. The participant was asked to touch this circle as fast as possible, then another yellow circle would light up. The time required by a participant to 96 indicated circles correctly was measured.

The overall vision score (vision total) was calculated by adding the z-standardized scores of all visual tests, (1) visual clarity of both eyes, (2) contrast sensitivity, (3) near-far quickness, (4) hand-eye coordination, and dividing this sum by the number of tests included (4).

### Procedure

The performance tests were conducted in a quiet and separated room. Executive functions were measured in a computerized four-task battery, lasting 45 min, each of the tasks starting with three practice trials, in which the participants had the opportunity to familiarize themselves with the tasks and ask questions. This ensured that each participant understood the task correctly. As a standard method in neuropsychological assessment, a fixed task order was used: (1) working memory, (2) cognitive flexibility, (3) inhibition, and (4) selective attention. The participants were asked to lean against the backrest of the chair and sit comfortably ensuring the same screen distance for each subject. Afterward, the visual functions were assessed in the following order: near-far quickness, contrast sensitivity, and visual clarity. These tests lasted 10 min. A detailed description of the test procedure is provided below. The data acquisition took place during the first 3 weeks of the seasons 2020/2021 or 2021/2022, respectively. The instructor of the test batteries was an experienced sports scientist, with a work experience of more than 5 years.

### Statistical analysis

An *a priori* power analysis was conducted using G*Power 3.1.9.6 to determine the sample size required for our hypotheses of medium correlations. Results indicate the required sample size of *n* = 49 to achieve 80% power at a significance criterion of α = 0.05. IBM SPSS Statistics 26.0.0 was used for the data analysis. Current recommendations to focus on estimation for best reporting and analysis practice were followed instead of conducting null-hypothesis significance tests (Cumming, 2014); effect sizes with 95% confidence intervals are reported. Not all variables can be assumed to be normally distributed, as indicated by the Shapiro-Wilk’s test (*p* < 0.05). Hence, correlations between the visual and cognitive performance of the subjects were assessed by Spearman’s correlation coefficient test. For an easier interpretation of our results, the scores of the tests were standardized so that a higher score always represents a better performance for each individual parameter.

Correlation coefficients (Spearman’s r) of 0.1, 0.3, and 0.5 represent small, moderate, and large effect size estimates, correspondingly (Cohen, 1988).

## Results

Partial correlation coefficients (Spearman’s r) with their 95% confidence intervals and the sample sizes of each correlation between the cognitive and visual performance parameters are depicted in Table 1. Generally, results with confidence intervals not including zero are meaningful as they depict reasonable evidence of a population effect (Cumming, 2014).

**TABLE 1:** Bivariate correlations between the executive and visual functions.

	Selective attention	Working memory	Cognitive flexibility	Inhibition	Executive function total
**Contrast sensitivity**					
Spearman’s *r*	0.224	0.258	0.095	0.122	**0.331**
CI	−0.05, 0.46	−0.01, 0.50	−0.18, 0.36	−0.15, 0.38	**0.04, 0.54**
*n*	53	53	53	53	**53**
**Visual clarity left eye**					
Spearman’s *r*	0.171	0.179	0.110	0.086	0.199
CI	−0.09, 0.41	−0.08, 0.42	−0.09, 0.41	−0.17, 0.33	−0.06, 0.43
*n*	59	59	59	59	59
**Visual clarity right eye**					
Spearman’s *r*	0.244	**0.288**	0.183	0.118	0.287
CI	−0.01, 0.47	**0.03, 0.50**	−0.07, 0.42	−0.14, 0.36	**0.02, 0.52**
*n*	59	**59**	59	59	**59**
**Visual clarity both eyes**					
Spearman’s *r*	0.256	**0.264**	0.213	0.123	**0.348**
CI	−0.01, 0.48	**0.01, 0.48**	−0.05, 0.44	−0.14, 0.37	**0.10, 0.55**
*n*	59	**59**	59	59	**59**
**Hand−eye coordination**					
Spearman’s *r*	**0.330**	0.181	0.161	0.100	**0.352**
CI	**0.08, 0.54**	−0.08, 0.42	−0.10, 0.40	−0.16, 0.35	**0.11, 0.56**
*n*	**59**	59	59	59	**59**
**Near−far−quickness**					
Spearman’s *r*	**0.505**	**0.379**	**0.370**	0.135	**0.602**
CI	**0.29, 0.67**	**0.14, 0.58**	**0.13, 0.57**	−0.13, 0.38	**0.41, 0.74**
*n*	**59**	**59**	**59**	59	**59**
**Visual total**					
Spearman’s *r*	**0.384**	**0.357**	0.254	0.124	**0.503**
CI	**0.13, 0.59**	**0.10, 0.57**	−0.17, 0.49	−0.15, 0.38	**0.27, 0.68**
*n*	**53**	**53**	53	53	**53**

CI = 95% confidence interval, boldface numbers highlighting CIs not including zero.

### Visual-total and executive functions

The visual-total displays a strong correlation with the executive function total [r_s_ (51) = 0.50 (95% CI: 0.27, 0.68)] and a moderate correlation with selective attention [r_s_ (51) = 0.38 (95% CI: 0.13, 0.59)] and working memory [r_s_ (51) = 0.36 (95% CI: 0.1, 0.57)].

### Contrast sensitivity and executive functions

Contrast sensitivity shows a meaningful moderate correlation with the executive function total score [r_s_ (51) = 0.33 (95% CI: 0.04, 0.54)]. Looking at the executive functions subgroups by themselves, no meaningful correlations can be found, but a trend toward a meaningful correlation is indicated for working memory [r_s_ (51) = 0.26 (95% CI: −0.01, 0.5)] and selective attention [r_s_ (51) = 0.22 (95% CI: −0.05, 0.46)].

### Visual clarity and executive functions

A moderate correlation is reported for the executive function total and the visual clarity of both eyes [r_s_ (57) = 0.35 (95% CI: 0.1, 0.55)] as well as the right eye [r_s_ (57) = 0.29, 95% CI: 0.02, 0.52)]. Additionally, working memory test performance show moderate correlations with the visual clarity of both [r_s_ (57) = 0.26 (95% CI: 0.01, 0.48)] eyes and the right eye [r_s_ (57) = 0.29 (95% CI: 0.03, 0.5)]. In contrast, no meaningful correlation is indicated for visual clarity of the left eye with the executive functions.

### Hand-eye coordination and executive functions

Hand-eye coordination showed a moderate correlation with executive function total [r_s_ (57) = 0.35 (95% CI: 0.11, 0.56] and selective attention [r_s_ (57) = 0.33 (95% CI: 0.08, 0.54)].

### Near-far quickness and executive functions

Strong correlations with near-far quickness were present for the cognition total score [r_s_ (57) = 0.60 (95% CI: 0.41, 0.74)] and selective attention [r_s_ (57) = 0.51 (95% CI: 0.29, 0.67)], whereby working memory [r_s_ (57) = 0.38 (95% CI: 0.14, 0.58)] and cognitive flexibility [r_s_ (57) = 0.37 (95% CI: 0.13, 0.57) correlate moderately.

## Discussion

The current study assessed the association between visual and executive functions in elite soccer players. In line with our first hypothesis, the results of this study indicate an overall relationship between all combined visual and all combined executive functions. Nevertheless, the individual linkages among certain visual skills and executive functions are quite specific.

One possible explanation for the overall vision-cognition association are the overlapping functional brain networks involved in visual and cognitive processes. Visual information received by the retina is initially processed in the superior colliculi which are not only involved in reflexive behaviors based on sensory input but also in higher cognitive processes like attention and decision-making since they have projections to forebrain structures like basal ganglia and the amygdala (Basso et al., 2021). The next stage of information processing within the visual pathway takes place in the visual thalamus whereby cognitive processes are assumed to already influence this visual processing even before the neuronal information has reached the visual cortex (O’Connor et al., 2002; Saalmann and Kastner, 2011). Moreover, it is also suggested that executive functions are arbitrated by more basic subcortical structures, which are also involved in aspects like visuospatial abilities (Sella et al., 2016; Saban et al., 2021).

Another aspect possibly explaining the correlation between visual and cognitive functions is that both processes rely on neuronal transmissions like neurotransmitters and white matter pathways (Forstmann et al., 2012; Jitsuishi et al., 2020) propagating chemical or electrical signals for the transduction of visual or cognitive information. Thus, a more efficient neuronal processing resulting in lower energy usage is linked to better visual and cognitive performance (Babiloni et al., 2010; Leisman et al., 2016). This neuronal efficiency is further based on the limited amount of the brain’s metabolic resources (Dietrich, 2006) potentially leading to the possibility that higher neural efficiency in visual processing frees up working space with a larger amount of energy that can be used for cognitive processing (Neubauer and Fink, 2009). The distinct correlations of the subfunctions underlying the large correlation between visual- and executive function total are further described based on the classification of visual functions by Burris et al. (2018). The first category “visual software” (near-far quickness, hand-eye coordination) showed the largest overall correlation with executive function total and all executive functions, especially selective attention which is partially in line with our second hypothesis. This may be originated in the inclusion of an array of physiological and psychomotor aspects in the near-far quickness skill like receptive visual processing, oculomotor control, and visual decision-making (Burris et al., 2018). Furthermore, the ability to quickly switch gaze focus between differing distances (i.e., near-far quickness) also resembles the ability to shift attention between task sets or strategies, i.e., cognitive flexibility (Miyake et al., 2000), and the attentional control-subcomponent of the working memory system (Chai et al., 2018).

The second category “visual hardware” including contrast sensitivity and visual clarity shows fewer correlations with smaller magnitudes compared to the “visual software” domain. Specifically, contrast sensitivity is only moderately related to the executive function total score and visual clarity of the right- and both eyes are only small to moderately correlated with working memory and the executive function total score. However, it is unclear why only the visual clarity of the right and both but not the left eye represents meaningful relations to executive functions. Previous literature suggests, that for the majority of people the right eye seems to be the dominant one (Lopes-Ferreira et al., 2013). Nevertheless, information on hemispheric side dominance for the visual system is scarce, thus the stronger correlations for right eye visual clarity compared to the left eye visual clarity, found in this study cannot be explained by previous findings.

Concerning our third hypothesis, working memory shows the most consistent correlation to the visual domains visual clarity of the right- and both eyes, near-far quickness, and the visual total score. These correlations could be based on the fact that working memory also contains a subcomponent called the visuospatial sketchpad (Baddeley, 2010). Thus, a more precise visual clarity might enhance the overall working memory performance using its visual-spatial component. Further, the underlying and shared neural pathways (Noudoost et al., 2021) and the influence of working memory on the circuitry transforming visual input (Roussy et al., 2021) could also be possible explanations for these correlations. It is also conspicuous that inhibition shows no meaningful relations with any of the included visual skills which may be originated in the application of a stop signal task with auditory stop signals. The usage of visual stop signals may have resulted in stronger relations to visual skills (Wu et al., 2019).

Regarding the aim of the study, the results may hint at the visual functions as a potential lever for possible performance enhancements of the executive functions as well as vice-versa. In particular, near-far quickness, working memory, and visual clarity represent the most promising starting points for a bidirectional regulation. However, future research is required to systematically prove the causal effect of executive functions’ performance enhancements by training visual skills as indicated by promising first evidence showing the transfer effects of training visual functions to perception and cognition (Wilkins and Appelbaum, 2020). Specifically, a randomized controlled trial investigating a 6-week vision training in a non-sport-specific context was able to demonstrate increases in cognitive functions in female volleyball players (Formenti et al., 2019). Additionally, improvements in short-term memory are also reported after stroboscopic visual training with students (Appelbaum et al., 2012).

Yet, it is also unclear whether those performance improvements appear the other way around as well, i.e., cognitive training improves visual functions. Preliminary evidence suggests that such transfer effects may be limited (Scharfen and Memmert, 2021a). Optimally, future studies investigating those relationships would track behavioral as well as neurophysiological changes.

### Limitations

By interpreting the results of this current study, some limitations have to be considered. The participants included were all-male elite soccer players. Previous studies have indicated that male and female athletes show different visual and cognitive performance skills (Burris et al., 2020). Furthermore, different sports disciplines have distinct requirement profiles for the athletes, thus a one-to-one transfer of results to other sports domains might be limited. Additionally, the sample size is not large enough to draw a final inference on the precise magnitude of the various correlations between visual and executive functions, as the 95% confidence intervals show a reasonable wide range. Moreover, the neurophysiological mechanism underlying the correlations named in this present study remains rather speculative.

## Conclusion

The results of this study indicate for the first time an overall relationship between performance data of distinct visual and executive functions. This representation of large vision-cognition correlations yields further insights concerning the interrelation of performance relevant aspects among each other expanding previous findings for example on the relation of cognition-motor connections (Scharfen and Memmert, 2019b,2021c). However, further studies are needed to investigate the underlying mechanisms of these correlations and the possible transfer effects of visual training interventions on cognitive functions and vice versa. First transfer effects from visual training to cognitive functions have been indicated in elite athletes (Formenti et al., 2019). However, future scientific attempts should specify the trainability of specific executive and visual functions with possible transfer effects. Especially the role and the trainability of visual skills like near-far quickness concerning possible transfer effects to executive functions should be examined based on the present results. In a second step, this could potentially lead to new training and scouting opportunities.

## Data availability statement

The raw data supporting the conclusions of this article will be made available by the authors, without undue reservation.

## Ethics statement

The studies involving human participants were reviewed and approved by the German Sports University Cologne. The patients/participants provided their written informed consent to participate in this study.

## Author contributions

AK and H-ES conceptualized and contributed to the analysis and interpretation of data of the work. AK, H-ES, and DM contributed to the design of the work. H-ES took part in the data acquisition. AK, H-ES, DM, ML, and JJ contributed to the drafting of this manuscript and discussed the results. All authors contributed to the article and approved the submitted version.
